# Voltage-gated potassium channels as important modulators of glucose-stimulated insulin secretion in pancreatic β-cells: insights from rodent and human studies

**DOI:** 10.3389/fendo.2026.1805302

**Published:** 2026-05-04

**Authors:** Jing-Jing Xing, Chen Chen

**Affiliations:** 1 School of Biomedical Sciences, University of Queensland, Brisbane, QLD, Australia; 2 Department of Medical Neuroscience, School of Medicine, Southern University of Science and Technology, Shenzhen, Guangdong, China

**Keywords:** glucose-stimulated insulin secretion (GSIS), insulin secretion, Kv channel, pancreatic β-cells, type 2 diabetes

## Abstract

Insulin secretion from pancreatic islet β-cells is governed by both metabolic and electrogenic pathways. The latter involves changes in membrane potential regulated by various potassium channels, including ATP-sensitive K^+^ (K_ATP_) channels, Ca^2+^-sensitive K^+^ (K_Ca_) channels, and voltage-dependent K^+^ (Kv) channels. Glucose metabolism elevates the ATP/ADP ratio, leading to the closure of K_ATP_ channels and subsequent membrane depolarization. Such depolarization opens voltage-dependent Ca^2+^ channels (vDCCs), allowing Ca^2+^ influx that triggers insulin release. The membrane is then repolarized through activation of K_Ca_ and Kv channels. Multiple K_Ca_ and Kv channel subtypes have been identified in insulin-secreting cells, with emerging evidence highlighting their significant modulatory roles in glucose-stimulated insulin secretion (GSIS). In this review, the current understanding and therapeutic potential of Kv channels in insulin secretion are discussed in relation to the structural features and physiological functions.

## 1 Introduction

Type 2 diabetes mellitus (T2DM) is a rapidly increasing chronic metabolic disease driven by the rising prevalence of obesity, which can lead to serious complications and high mortality (1). The current strategies for managing obesity are summarized in **Table 1**. T2DM is currently a global health crisis, especially in developing countries. In 2021, it was estimated that 536 million adults had diabetes (more than 90% of which were T2DM), which brought $966 billion in annual medical costs (19). Current treatments for T2DM involve medications or insulin to maintain blood glucose levels as close to normal as possible. Most antidiabetic drugs lower blood glucose levels (including fasting levels) either by stimulating endogenous insulin secretion or by improving insulin sensitivity (20). Metformin, a first-line therapy, primarily reduces fasting blood glucose by improving hepatic insulin sensitivity and decreasing glucose production. Sulfonylureas serve as second-line therapy for T2DM by directly stimulating insulin secretion through closure of ATP-sensitive potassium (K_ATP_) channels (20, 21). Thiazolidinediones, as insulin sensitizers, primarily treat T2DM by enhancing insulin action in skeletal muscle, adipose tissue, liver, and cardiac muscle (20). Glucagon-like peptide 1 receptor (GLP-1) agonists enhance insulin secretion and promote β-cell proliferation in the treatment of diabetes (primarily studied in rodent models).

**Table 1:** Current therapies of obesity.

Weight management medication	Effectiveness	Mechanisms	References
Orlistat	10% within 6 months, less effective	• Lipase inhibitors • Preventing some of the fat in foods eaten from being absorbed in the intestines	(2–4)
Phentermine–topiramate	10% and 20% at the highest dose (15/92mg)	• A mix of two medications: phentermine, which lessens your appetite, and topiramate, which is used to treat seizures or migraine headaches • May make you less hungry or feel full sooner	(5, 6)
Naltrexone–bupropion	5% weight loss within 6 months	• A mix of two medications: naltrexone, which is used to treat alcohol and drug dependence, and bupropion, which is used to treat depression or help people quit smoking • May make you feel less hungry or full sooner	(7–9)
Liraglutide	5% weight loss in 56 weeks in diabetes	• Mimics a hormone called glucagon-like peptide-1 (GLP1) that targets areas of the brain that appetite and food intake • At a lower dose under a different name, Victoza, this drug is FDA-approved to treat type 2 diabetes	(10–12)
Semaglutide	The initial dose is 0.25 mg, once weekly for 4 weeks. The dose is increased in 4-week intervals (over a total of 16 weeks) until a dose of 2.4 mg per week is reached.	• Mimics a hormone called GLP-1 that targets areas of the brain that regulate appetite and food intake. • Under different names and dosages, this drug is FDA- approved to treat type 2 diabetes as an injectable medication (Ozempic) and as an oral pill (Rybelsus)	(13, 14)
Setmelanotide	For patients 12 years and older, the starting dose is 2 mg daily for 2 weeks before titrating. In patients aged 6 to 11 years, the starting dose is 1 mg daily for 2 weeks.	• Activates pathways in the brain to promote weight loss by decreasing appetite and food intake while increasing the number of calories the body uses	(15, 16)
Tirzepatide	5 mg once weekly. This can be titrated up to 15 mg once weekly based on individual response and tolerability	• Mimics two hormones, glucose-dependent insulinotropic polypeptide (GlP) and GLP-1 to target areas of the brain that regulate appetite and food intake. • Under a different name, this drug is FDA-approved to treat type 2 diabetes as an injectable medication	(17, 18)

Insulin is only secreted by pancreatic β-cells and is the only peptide hormone that effectively reduces blood sugar in the human body. Insulin secretion is regulated by blood glucose levels. When the blood glucose concentration rises, glucose enters the cytoplasm of β-cells via glucose transporters (Gluts). Metabolism of glucose in mitochondria in β-cells results in an increase in ATP production from ADP, which in turn increases the ATP: ADP ratio. Such increased ratio leads to the closure of the ATP-sensitive potassium (K_ATP_) channel. Closure of KATP channels reduces K^+^ efflux, leading to membrane depolarization. Membrane depolarization results in an influx of calcium ions through voltage-gated Ca^2+^ channels (Cav), resulting in an increase in cytoplasmic free Ca^2+^ (Ca^2+^ i) levels. Intracellular Ca²^+^ triggers exocytosis of insulin-containing secretory granules, resulting in the release of insulin. When K_ATP_ channels are closed, the secretion of insulin is triggered. KATP channel inhibitors, such as sulfonylureas, are therefore used in the treatment of T2DM. By directly closing KATP channels, sulfonylureas stimulate insulin secretion independently of circulating glucose levels and the associated rise in ATP/ADP ratio. Consequently, this glucose-independent mechanism can lead to hypoglycemia in patients receiving sulfonylurea therapy (22).

The voltage-gated channels involved in the rising phase of the action potentials (AP) of β-cells are mainly the Ca^2+^ and Na^+^ channels (23). Continued depolarization activates the voltage-gated K^+^ channels (Kv). The GSIS ends therefore with the repolarization of the cell membrane back to resting potential (RP). The Kv channels are the major driving channels to repolarize the β-cell plasma membrane. In the last few decades, significant progress has been made toward elucidating the mechanism of voltage-gated K^+^ channels. Kv channels are present in most cell types in maintaining the resting potential of the cells. Studies of Kv channel function and regulation are gaining momentum and have benefited from the pooling of resources ranging from biophysics to genomics. More than 40 human genes encode different K^+^ channel subunits, and mutations of a number of these genes have been found to cause dysfunction of the heart, kidney, pancreas, and central nervous system (24). Future studies are likely to further our understanding of how these Kv channels are organized and functionally integrated in a cell, and how these channels may be modulated in the treatment of diseases.

Kv2.1 inhibition was first shown to enhance insulin secretion in human islets (25). From then on, increasing attention has been focused on this Kv channel, which regulates membrane repolarization, with effects on AP duration and frequency (26, 27). Activation of Kv repolarizes β-cell action potential during a glucose stimulation to limit Ca^2+^ entry and insulin secretion. Kv channels normally open after membrane depolarization, which allows K^+^ efflux to repolarize the β-cell plasma membrane. Some studies have found that the use of some Kv channel inhibitors (such as Kv2.1 and Kv11.2) may prolong the depolarization phase of the β-cell action potential, thereby increasing insulin secretion (28, 29). Thus, Kv channels have the prospect of being a new therapeutic target for T2DM.

While K_ATP_ channels serve as the primary glucose sensors initiating depolarization, and Cav channels drive the Ca²^+^ influx essential for exocytosis, Kv channels primarily function as repolarizing modulators that limit AP duration and Ca²^+^ entry, thereby fine-tuning the amplitude and duration of insulin secretion, particularly in the sustained phase. In this literature review, the structure and function of Kv channels expressed primarily in human and mouse pancreatic β-cells are described. In addition, this review discusses how these Kv channels regulate insulin secretion and the potential role of Kv channels in T2DM. This review proposes that, in contrast to KATP channels which serve as the primary glucose sensor initiating the triggering phase of GSIS, Kv channels function as “phase-specific rheostats” that fine-tune β-cell excitability, action potential (AP) dynamics, and Ca²^+^ oscillations, thereby determining the amount, speed, and duration of the sustained (second) phase of insulin secretion.

## 2 Structure and assembly of the Kv channel

**Figure 1:**
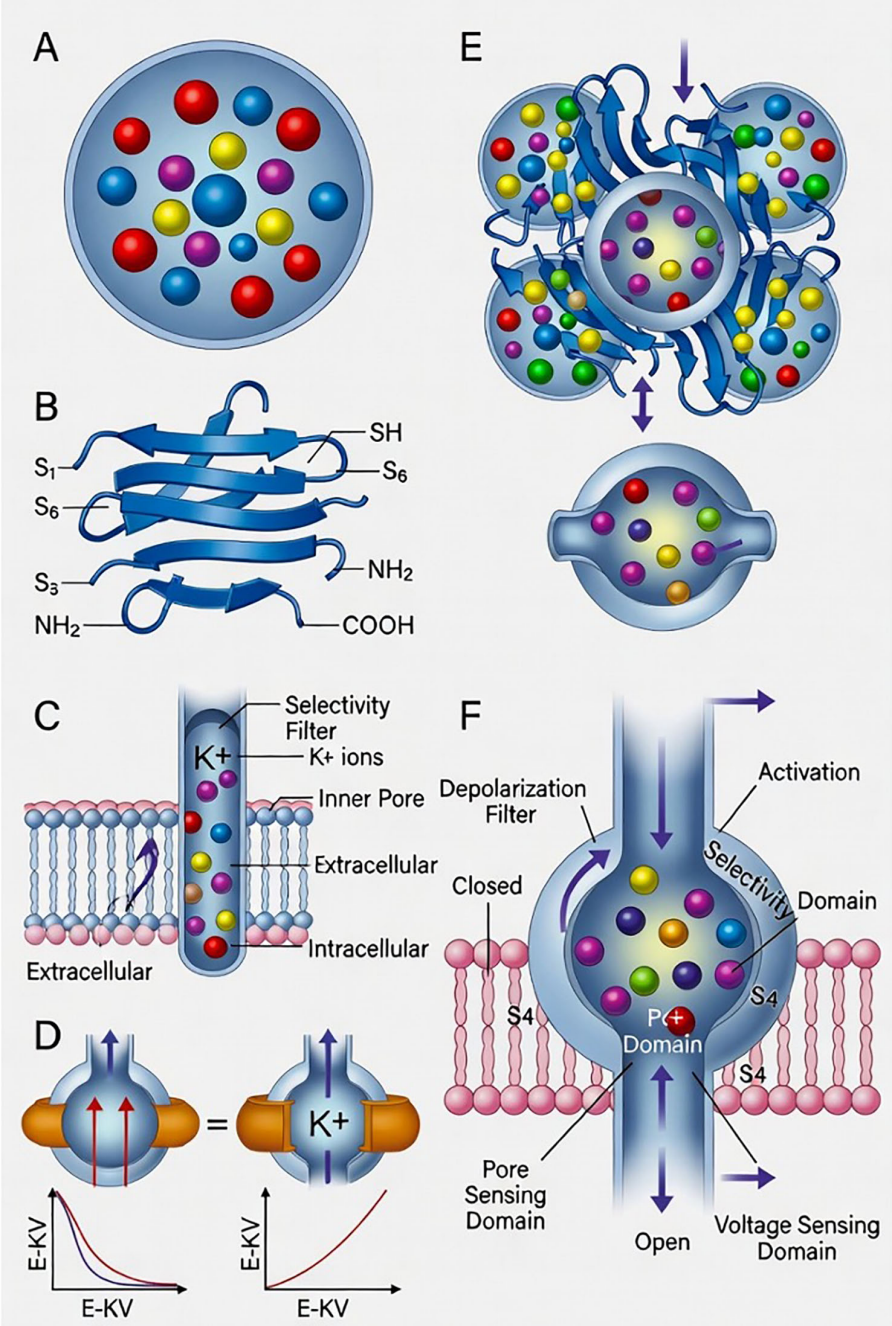
Structure of Kv channels and regulation on insulin secretion. **(A)** Rodent and human islet of Langerhans cell distribution. **(B)** Structure diagram of the α-subunit in the cell membrane. The α-subunit consists of six transmembrane segments (S1 to S6) and an N-terminal and a long C-terminal cytoplasmic domain. **(C)** Structure diagram of the Kv channel in the cell membrane. **(C)** Kv channels are tetramers formed by four α-subunits. **(D)** The Kv channel regulates currents changes on the membrane. **(E)** Kv channel efficiency on insulin secretion in normal conditions. **(F)** Closed Kv channel efficiency on insulin secretion.

Kv channels are tetramers consisting of four α-subunits (30). Each α-subunit contains six transmembrane segments (S1 to S6), an N-terminal domain, and a long C-terminal cytoplasmic domain (**Figure 1**). The S1 to S4 transmembrane segments form the voltage-sensing domain, which detects membrane potential and trigger the opening and closing of the channel (31). The S4 transmembrane segment has many positively charged amino acid residues and is the primary voltage sensor responsible for generating gating currents (32). The S5 and S6 transmembrane segments form the pore structure. A conserved selectivity filter sequence (glycine–tyrosine–glycine or glycine–phenylalanine–glycine) is located in the pore loop (P-loop) between S5 and S6, conferring K^+^ ion selectivity (33).

Certain α-subunits in the Kv channel family (Kv5, Kv6, Kv8, Kv9) are electrically silent (KvS) and do not form functional homotetramers upon depolarization (34). Kv2 subunits can only function by forming heterotetramers (KvS/Kv2) (35). The formation of heterotetramers between KvS and Kv2 is determined by the T1 domain (36). Co-expression experiments showed that Kv8.1 forms heterotetramers with Kv2.1 but not Kv1.3 (37). Only after replacing the T1 domain of Kv8.1 with that of Kv1.3 could Kv8.1 form heterotetramers with Kv1.3 (37). A conserved acidic motif (CDD) in the T1 domains of KvS and Kv2 subunits is critical for heterotetramerization (31–36). Heterotetramers of KvS and Kv2 exhibit distinct biophysical properties compared with Kv2 homotetramers (35). Thus, KvS subunits act as regulatory modulators of Kv2, primarily by altering current amplitude (31). Due to cell- and subtype-specific differences, these aspects are beyond the scope of this review.

In addition to forming functional homotetramers, Kv subunits also form heterotetramers with other subunits in the same family. For example, α-subunits in the Kv1 family form heterotetramers with each other. Heterotetramers within the same family (e.g., Kv1) increase channel diversity, with distinct kinetics and pharmacology compared with homotetramers (38). For example, Kv1.3 and Kv1.5 may form different heterotetramer channels in different ratios of 1:1, 1:3, and 3:1, and the biophysical properties of these heterotetramers are also different (39). The biophysical characteristics of heterotetramers composed of distinct α-subunits within the same family are intricate and fall outside the scope of this review.

## 3 Types of Kv channels/currents in pancreatic β-cells

The primary function of Kv channels is to repolarize the membrane potential. Depolarization of the cell membrane activates voltage-gated Kv channels. Upon activation, Kv channels mediate outward K^+^ efflux, which repolarizes the membrane potential and restores it to the resting state. Since the initial identification of the Shaker locus gene encoding a Kv channel in Drosophila locus Shaker (40), a total of 40 Kv channel genes have been identified to date. These 40 Kv channels are classified into 12 subfamilies (Kv1-Kv12) (41–43), which are shaker-related family (Kv1), Shab-related family (Kv2), Shal-related family (Kv3), Shaw-related family (Kv4), modifiers (Kv5, Kv6, Kv8, Kv9), KVLQT family (Kv7.1), KQT2 family (Kv7.2-Kv7.5), EAG family (Kv10), ERG family (Kv11), and EIK family (Kv12) (**Table 2**). Different Kv channels carry different Kv currents. Based on their electrophysiological properties, Kv channels are primarily classified into two main types: delayed rectifier Kv channels and A-type Kv channels.

**Table 2:** Classification of Kv channels and their distribution in various tissues.

Family	Gene	Alias	K_v_ current	Channel distribution	Reference
Shaker-related family	KCNA1	K_v_1.1	Delayed rectifier	Brain, heart, retina, skeletal muscle, islets	(44–48)
	KCNA2	K_v_1.2	Delayed rectifier	Brain, heart, retina, skeletal muscle, islets	(49)
	KCNA3	K_v_1.3	Delayed rectifier	Brain, lung, islets, T-cell, B-cell	(49–51)
	KCNA4	Kv1.4	A-type	Brain, heart, islets	(49)
	KCNA5	Kv1.5	Delayed rectifier	Aorta, colon, kidney, brain, skeletal muscle	(49, 52)
	KCNA6	Kv1.6	Delayed rectifier	Brain, lung, colon, heart,	(49)
	KCNA7	Kv1.7	Delayed rectifier	islets, skeletal muscle, heart	(49)
	KCNA10	Kv1.8	Delayed rectifier	Kidney, brain, heart	(49)
Shab-related family	KCNB1	Kv2.1	Delayed rectifier	Brain, lung, islets, eye, skeletal	(49, 53)
	KCNB2	Kv2.2	Delayed rectifier	Brain, islets, smooth muscle	(49, 54)
Shaw-related family	KCNC1	Kv3.1	Delayed rectifier	Brain, lung, skeletal muscle	(49)
	KCNC2	Kv3.2	Delayed rectifier	Brain, islets	(49)
	KCNC3	Kv3.3	A-type	Brain	(49)
	KCNC4	Kv3.4	A-type	Brain	(49)
Shal-related family	KCND1	Kv4.1	A-type	Brain, heart, lung, islets	(49)
	KCND2	Kv4.2	A-type	Brain	(49)
	KCND3	Kv4.3	A-type	Heart, brain, smooth muscle, liver, islets, prostrate, and kidney	(49, 55)
Modifiers	KCNF1	Kv5.1	None	Heart, brain, skeletal muscle, liver	(49)
	KCNG1	Kv6.1	None	Heart, brain, smooth muscle, kidney	(49)
	KCNG2	Kv6.2	None	Brain	(49, 56)
	KCNG3	Kv6.3	None	Brain	(49)
	KCNG4	Kv6.4	None	Brain, liver	(49)
KvLQT	KCNQ1	Kv7.1	Delayed rectifier	Heart, kidney, lung, stomach, small and large intestine, kidney, and pancreas	(49, 57)
KQT2	KCNQ2	Kv7.2	Delayed rectifier	Brain, heart	(49)
	KCNQ3	Kv7.3	Delayed rectifier	Brain	(49)
	KCNQ4	Kv7.4	Delayed rectifier	Brain, heart, and skeletal muscle	(49, 58)
	KCNQ5	Kv7.5	Delayed rectifier	Brain, skeletal muscle	(49)
Modifiers	KCNv1	Kv8.1	None	Brain, kidney	(49)
	KCNv2	Kv8.2	None	Lung, liver	(49)
	KCNS1	Kv9.1	None	Brain	(49)
	KCNS2	Kv9.2	None	Brain, retina	(49)
	KCNS3	Kv9.3	None	Brain, kidney, heart, lung	(49)
EAG1	KCNH1	Kv10.1	Delayed rectifier	Brain	(49)
EAG2	KCNH5	Kv10.2	Delayed rectifier	Brain	(49)
ERG1	KCNH2	Kv11.1	Not established	Heart, brain, kidney, liver, lung	(49)
ERG2	KCNH6	Kv11.2	Not established	Brain, islets	(49, 59)
ERG3	KCNH7	Kv11.3	Not established	Brain	(49)
EIK1, EIK3	KCNH8	Kv12.1	Delayed rectifier	Brain, lung, colon	(49)
EIK2	KCNH3	Kv12.2	Not established	Brain, lung	(49)
EIK1	KCNH4	Kv12.3	Delayed rectifier	Brain, lung	(49)

When the membrane potential is depolarized, the membrane currents activate rapidly and then deactivate, whereas delayed rectifier K^+^ currents activate slowly and then slowly deactivate (60). This classification (delayed rectifier versus A-type) does not correspond to specific Kv subfamilies but rather depends on the properties of individual α-subunits.

For instance, Kv1.1 functions as a delayed rectifier channel, whereas Kv1.4 produces an A-type current. Although both Kv1.1 and Kv1.4 belong to the Kv1 subfamily, their functional properties differ due to structural variations in their α-subunits. For example, Kv1.4 contains an N-terminal inactivation domain (“ball-and-chain” mechanism) that confers rapid inactivation, a hallmark of A-type currents. Thus, functional classifications (delayed rectifier versus A-type) transcend subfamily boundaries and are better defined by the intrinsic properties of individual α-subunits.

## 4 Kv channels in rodent pancreatic islet β-cells

In mouse β-cells, the pore-forming α-subunits KCNB1 (Kv2.1), KCNH1 (Kv10.1), KCNH2 (Kv11.1), and KCNH6 (Kv11.2) are expressed at high levels (61). Among these, Kv2.1 knockout mice exhibit elevated serum insulin levels and reduced fasting blood glucose, indicating that Kv2.1 channels contribute to β-cell membrane repolarization (53). Furthermore, Kv2.1-knockout β-cells show an ~83% reduction in Kv current compared with controls, suggesting that the Kv2.1 channel is the predominant Kv channel regulating β-cell membrane repolarization (53). While most other Kv channels contribute minimally to β-cell membrane repolarization, certain channels (e.g., from the Kv11/ERG family) do play a role. For instance, KCNH6 knockout mice show elevated intracellular Ca²^+^ concentrations and enhanced insulin secretion in islet cells (59). In addition to delayed rectifier K^+^ currents, mouse β-cells exhibit TEA-sensitive delayed rectifier Kv currents and 4-AP-sensitive A-type K^+^ currents (62). The A-type K^+^ currents observed in mouse β-cells may arise from Kv3.2 and/or Kv3.3 channels (**Table 3**).

**Table 3:** Kv channel in rat pancreatic islet β-cell.

Family	Gene	Alias	Reference
Shaker-related family	KCNA1	Kv1.1	(63)
	KCNA2	Kv1.2	(61)
	KCNA7	Kv1.7	(64)
Shab-related family	KCNB1	Kv2.1	(65)
	KCNB2	Kv2.2	(61)
Shaw-related family	KCNC2	Kv3.2	(66)
	KCNC3	Kv3.3	(61)
Shal-related family	KCND1	Kv4.1	(61)
KvLQT	KCNQ1	Kv7.1	(67)
EAG1	KCNH1	Kv10.1	(61)
EAG2	KCNH5	Kv10.2	(61)
ERG1	KCNH2	Kv11.1	(68)
ERG2	KCNH6	Kv11.2	(59)

## 5 Kv channels in human pancreatic islet β-cells

In human β-cells, the pore-forming α-subunits KCNB2 (Kv2.2), KCNA5 (Kv1.5), KCNA6 (Kv1.6), KCNC3 (Kv3.3), KCND3 (Kv4.3), KCNQ2 (Kv7.2), and KCNH2 (Kv11.1) are expressed at high levels (61). Compared with mouse β-cells, where Kv2.1 is predominant as the main delayed rectifier channel, Kv2.1 does not appear to be the primary contributor to delayed rectifier currents in human β-cells. Instead, Kv2.2 (KCNB2) is expressed at substantially higher levels than Kv2.1 (69). Accordingly, Kv2.2, along with other channels (e.g., Kv1.6 and possibly BK channels), plays a major role in mediating repolarization of the β-cell membrane in humans (61). Although Kv2.1 is not a major component of the delayed rectifier outward K^+^ current in human β-cells, it can promote insulin exocytosis independently of its electrical function, likely through channel clustering and direct interaction with SNARE proteins such as syntaxin 1A (25). Silent subunits Kv6.2 and Kv9.3 are also expressed in human pancreatic β-cells (70) (**Table 4**). These subunits cannot form functional homotetrameric channels but can co-assemble with Kv2 family subunits to form heterotetrameric channels, thereby modulating the biophysical properties of Kv2 currents (34), thus regulating the biophysical properties of Kv2. However, whether Kv6.2 and Kv9.3 assemble with Kv2 subunits to regulate Kv2 currents in human β-cells remains unknown. Additionally, the Kv4.3 gene (KCND3) is expressed at relatively high levels in human β-cells, suggesting that it may serve as a major contributor to A-type currents in these cells.

**Table 4:** Kv channel in human pancreatic islet β-cell.

Family	Gene	Alias	Reference
Shaker-related family	KCNA5	Kv1.5	(61)
	KCNA6	Kv1.6	(61)
Shab-related family	KCNB1	Kv2.1	(25)
	KCNB2	Kv2.2	(69)
Shaw-related family	KCNC3	Kv3.2	(70)
Shal-related family	KCND3	Kv4.3	(61)
Modifiers	KCNG2	Kv6.2	(70)
KQT2	KCNQ2	Kv7.2	(61)
Modifiers	KCNS3	Kv9.3	(70)
ERG1	KCNH2	Kv11.1	(61)
ERG2	KCNH6	Kv11.2	(61)

## 6 Kv channels function in insulin secretion

Pancreatic β-cells express approximately 50 different types of ion channels (71). Different Kv channels are selectively expressed in various tissues (**Table 2**). The Kv channels expressed in human and rodent (particularly mouse) islets have received considerable attention and are discussed in detail (70). Here, we summarized the major Kv channels in pancreatic islet β-cells and discuss how these diverse Kv channels regulate insulin secretion.

GSIS comprises two main pathways: the triggering pathway and the amplifying pathway (72). For the triggering pathway, the process is well-documented (73), which includes three process (1): the uptake of glucose is metabolized in mitochondria by the tricarboxylic acid cycle, inducing the increased ratio of ATP/ADP; (2) the KATP channel in islet β-cells senses the ratio changes and is closed, which in turn results in depolarization; and (3) depolarization in the β-cell membrane stimulates the opening of voltage-operated Ca^2+^ channels and accelerates the inflow of Ca^2+^ into cytoplasm and activates the exocytoxicity of insulin. This process primarily involves readily releasable granules docked at the plasma membrane, which fuse rapidly with the membrane upon Ca²^+^ influx, corresponding to the well-known first phase of insulin secretion (74). The amplifying pathway involves the recruitment of additional granules from intracellular reserves following depletion of the readily releasable pool, sustaining insulin secretion over extended periods (the second phase). Although the amplifying pathway has gained increasing acceptance, it primarily involves KATP-independent mechanisms.

**Figure 2:**
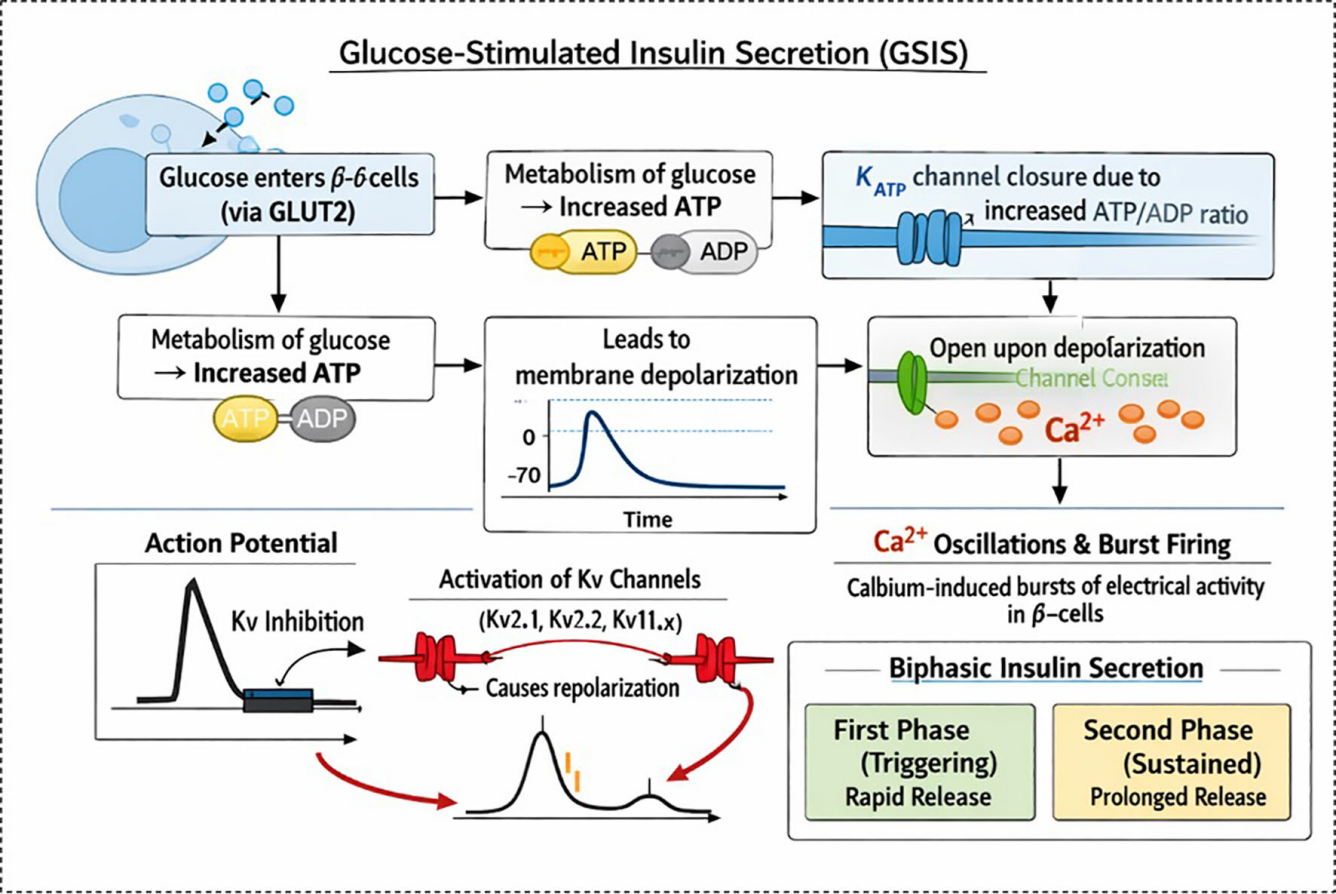
Mechanism of GSIS in pancreatic β-cells. The schematic illustrates the step-by-step process of glucose-stimulated insulin secretion in β-cells. Glucose enters the β-cell via the GLUT2 transporter and undergoes metabolism, leading to an increased ATP/ADP ratio. This causes closure of ATP-sensitive potassium (KATP) channels, resulting in membrane depolarization. Depolarization opens voltage-gated calcium (Ca²^+^) channels, allowing Ca²^+^ influx, which triggers Ca²^+^ oscillations and burst firing. This electrical activity activates voltage-gated potassium (Kv) channels (Kv2.1, Kv2.2, and Kv11.x), which promote repolarization and shape the action potential. The overall process leads to biphasic insulin secretion: a rapid first phase (triggering and rapid release of insulin granules) followed by a sustained second phase (prolonged insulin release).

Following KATP channel closure and initial membrane depolarization, voltage-gated Ca²^+^ channels (Cav) open to generate the rising phase of the action potential. Kv channels are subsequently activated to repolarize the membrane, thereby limiting Ca²^+^ influx per spike while permitting rhythmic bursting and oscillatory Ca²^+^ patterns that are essential for sustained second-phase GSIS (**Figure 2**). In rodent β-cells, Kv2.1 constitutes the major delayed-rectifier current and sets the burst frequency; its inhibition or ablation prolongs the AP plateau duration, increases the Ca²^+^ oscillation amplitude/frequency, and enhances second-phase secretion (Section 3). In human β-cells, Kv2.2 and Kv11.1 (HERG) play more prominent roles, with Kv11.1 blockade increasing the firing frequency and altering Ca²^+^ oscillations (Section 6). Recent studies in human and rodent models confirm that Kv2.2 regulates repetitive AP firing and inhibits (e.g., via the PGE2-EP2/4-PKA pathway) the firing rate and GSIS (75). Thus, Kv channels do not merely terminate APs but actively sculpt burst firing patterns and Ca²^+^ dynamics, converting transient depolarization into pulsatile insulin release.

In this minireview, we highlight the following three concepts. Firstly, Kv channels link basal insulin levels more directly to membrane excitability than KATP channels. Tonic/low-level activity of delayed-rectifier Kv channels (especially Kv2.x and Kv11.x) helps set the subthreshold resting membrane potential and basal excitability, thereby modulating low-level insulin release even at sub-stimulatory glucose concentrations (2.8–5 mM). In contrast, KATP closure is the dominant, glucose-dependent switch for GSIS initiation. Secondly, Kv channels determine not only the amount but also the speed and duration of the GSIS response. By controlling repolarization kinetics, Kv channels regulate (i) AP plateau duration (affecting Ca²^+^ influx per spike → secretion amount), (ii) inter-burst intervals (influencing oscillation frequency → response speed), and (iii) overall burst duration (prolonging the active phase → secretion duration). This is evidenced by Kv2.1 knockout mice (~83% reduction in Kv current with elevated serum insulin; Section 3) and KCNH6 (Kv11.2) studies showing prolonged AP duration and biphasic dysregulation (Sections 6 and 9). Third is species- and subtype-specific fine-tuning with non-electrical functions. Rodent β-cells rely predominantly on Kv2.1 for repolarization, whereas human β-cells show greater contributions from Kv2.2 and Kv11.1, KCNH6 (76), plus Kv2.1’s SNARE-clustering role in exocytosis independent of ion flux (Section 6 (25), (summarized in **Table 5**). Recent human islet data further emphasize Kv2.2’s role in regulating repetitive firing and PGE2-mediated inhibition of GSIS (75).

**Table 5:** Summary of Kv channel subtypes in pancreatic β-cells: expression (rodent vs. human) and functional roles in GSIS.

Kv subtypes	Current type	Expression levels (Rodent β-cells / Human β-cells)	Key function roles
Kv 2.1 (KCNB1)	Delayed rectifier	Rodent predominant (60–85%) Human Medium/Low	Repolarization
Kv 2.1 (KCNB2)	Delayed rectifier	Medium High	Repolarization Indirect via β-cells
Kv 2.1 (KCND3)	A-Type (rapid/fast inactivation)	Low/Medium High	Burst frequency, response speed
Kv 6.2 (KCNG2)	Electrically silent (modulate Kv2)	Rodent Low Human Expressed	Altering Kv2 kinetics
Kv 9.3 (KCNS3)	Electrically silent (modulate Kv2)	Rodent Low Human Expressed	Altering Kv2 kinetics

## 7 Kv alpha subunits and insulin secretion

Voltage-gated potassium (Kv) channels, particularly their α-subunits, play a crucial role in shaping the electrical activity of pancreatic β-cells and regulating insulin secretion. The pore-forming α-subunits assemble into functional tetrameric channels, whereas certain modulatory (silent) α-subunits associate with members of the Kv1–4 families to alter channel properties (77–80). These α-subunits determine key biophysical characteristics, including gating kinetics, conductance, and voltage sensitivity (81).

Kv channels generate outward delayed rectifier potassium currents that contribute to glucose-stimulated insulin secretion (GSIS). Voltage-dependent outward K^+^ currents account for approximately 15%–20% of the total voltage-dependent outward currents in β-cells (60).

Subunits of the Kv1 family primarily regulate the initiation and shaping of action potentials (APs). Several members, such as Kv1.1, Kv1.2, Kv1.4, and Kv1.6, are predominantly expressed in the brain (82), where most studies have focused on their role in controlling neuronal excitability rather than in islet β-cells (83). In Kv1.3 knockdown mice fed a high-calorie diet, animals gain weight but exhibit low blood insulin levels despite normal glucose levels (84). Inhibition of Kv1.3 enhances glucose uptake and GLUT4 translocation in adipose tissue and skeletal muscle, indicating increased peripheral insulin sensitivity (85). Inhibition of human Kv1.5 (hKv1.5) is reported to increase arrhythmia susceptibility, with fewer studies on its role in pancreatic β-cell excitability, possibly due to its higher expression in pulmonary arteries, brain, and skeletal muscle (52). Treatment of Wistar rats with conkunitzin-S1 (Conk-S1; 100 nmol/kg), a selective Kv1.7 blocker, enhances GSIS without affecting basal glucose levels (64).

Both Kv2.1 (KCNB1) and Kv2.2 (KCNB2) mediate outward potassium currents. In human islets, Kv2.2 expression is substantially higher than that of Kv2.1; however, only Kv2.1 facilitates insulin exocytosis—likely through channel clustering and direct interaction with SNARE proteins (25), Selective knockdown of Kv2.2 does not impair depolarization-induced exocytosis in human β-cells. Kv2.1 expression is reduced in T2D islets, yet its clustering promotes; these differences may arise from cell-type-specific localization within the islets (86). In mouse islets, Kv2.2 silencing increases somatostatin expression without altering insulin secretion, consistent with its localization in δ-cells (at least in rodents). In mice lacking somatostatin receptor 5, the Kv2 blocker GxTX-1E stimulates insulin secretion and improves glucose tolerance (54).

Kv7.1 (KCNQ1) is expressed in rodent pancreatic islets (87). Inhibition of Kv7.1 enhances insulin secretion in MIN6 cells, whereas elevated Kv7.1 expression in rat pancreatic β-cells reduces insulin secretion, highlighting potential clinical implications (67). In isolated cultured islets, the Kv7.1 inhibitor chromanol 293B enhances GSIS without affecting basal insulin secretion; it also improves glucose tolerance and insulin response in ICR mice (88). Clinically, patients with KCNQ1 dysfunction exhibit increased insulin release and lower plasma glucose upon oral glucose challenge (89). However, loss-of-function mutations in KCNQ1 cause long QT syndrome (LQTS), a cardiac condition that can lead to rapid, chaotic heartbeats (89, 90). Thus, while Kv7.1 inhibition may delay T2D progression, it carries potential cardiac side effects.

The Kv5, Kv6, Kv8, and Kv9 subunits are electrically silent and cannot form functional homotetrameric channels. Instead, they form heterotetrameric channels with Kv2 family members (particularly Kv2), modulating Kv2 current properties in a tissue-specific manner (91). In the pancreatic β-cell line MIN6, overexpression of KCNQ1 (Kv7.1) significantly increases Kv7.1 and total K^+^ currents, diminishing the effects of tolbutamide (a KATP inhibitor) and pyruvate (a glycolysis product) on insulin secretion (92). However, the role of KCNQ1 remains controversial: Homozygous KCNQ1 knockout mouse islets show no change in insulin secretion compared with the wild type (93), whereas siRNA-mediated silencing of KCNQ1 enhances insulin secretion in diabetic human β-cells (94). Given the low expression of KCNQ1 in both human and mouse β-cells, its deletion is unlikely to substantially reduce K^+^ currents, leaving its contribution to β-cell function unclear.

Kv11 (ERG) channels, including KCNH2 (Kv11.1/HERG1) and KCNH6 (Kv11.2), are expressed in rat insulinoma INS-1 cells and rodent β-cells, where they contribute to membrane repolarization. Inhibition of ERG1–3 channels by E4031 increases insulin secretion in INS-1 cells (95), and ERG1 blockade elevates intracellular Ca²^+^ in mouse β-cells. In human β-cells, KCNH2 (Kv11.1/HERG1) is expressed at levels more than twofold higher than KCNB2 (Kv2.2) (96). Blockade of HERG1 with WAY-123,398 increases the firing frequency in human β-cells and, at high glucose concentrations, significantly enhances insulin secretion in human islets, indicating that HERG channels regulate action potential repolarization and contribute to insulin secretion (97). A similar study used the siRNA approach in knockdown KCNH6 (Kv11.2) rodent INS-1 β-cells, shown apparently to have elevated insulin secretion at a high glucose level. This result is consistent with the study conducted in electrophysiological properties of Kv11.2; KCNH6 in plasmids encoded with human wild type and mutant were transfected into the HEK293 cell. Although the KCNH6 mutant and wild type have similar protein expression levels, the KCNH6 mutant had a lower step-current density, lower tail-current density, and more rapid activation than the wild type. In an additional study of Kv current in both KCNH6 knockout and knock-in mouse, Kv current was shown to be significantly decreased. Within that, there were no changes in the amplifying of the action potential, but the prolonged duration of the action potential led to cellular excitability. Another possibility of KCNH6 contribution in insulin secretion is its localization; the wild-type KCNH6 protein is expressed at the cell plasma membrane, whereas the KCNH6 mutant localizes near the cell nucleus (59).

## 8 Kv channels β subunits in insulin secretion

While the α subunits of Kv channels, such as Kv2.1, form the primary pore-forming components and dominate the delayed rectifier currents responsible for action potential repolarization in pancreatic β-cells, the β subunits play a more auxiliary yet significant role in modulating channel heterogeneity, kinetics, and function. However, compared with α subunits, the contributions of β subunits to GSIS are less extensively characterized and appear less central to the core electrophysiological regulation of insulin release. Their importance lies primarily in fine-tuning Kv channel properties rather than driving the fundamental repolarizing currents. Nonetheless, emerging evidence suggests they influence β-cell excitability and secretion under specific conditions, warranting discussion in parallel with α subunits for a comprehensive understanding of Kv channel diversity.

Kv β subunits, including the cytoplasmic Kvβ1–3 family (encoded by KCNAB1-3), are expressed in human and rodent pancreatic islets and can associate with the N-terminus of certain α subunits, particularly those in the Kv1 family, to regulate channel trafficking, expression, and gating properties (60, 98). These β subunits possess aldo-keto reductase activity, conferring redox sensitivity to the channels, which may allow β-cells to adapt Kv currents in response to metabolic states or oxidative stress. Although Kv2.1 is the predominant α subunit mediating 60%-85% of the voltage-dependent outward K^+^ currents in β-cells, Kvβ subunits do not directly interact with Kv2.x channels but may indirectly influence overall Kv heterogeneity through interactions with other expressed α subunits like Kv1 or Kv3 family members (39). For instance, in rodent β-cells, Kvβ subunits can enhance inactivation kinetics or alter voltage dependence, potentially prolonging action potentials and enhancing Ca²^+^ influx if disrupted, although direct impacts on GSIS remain underexplored.

In addition to the Kvβ1–3 family, the Kv channel-interacting proteins (KChIPs), such as KCNIP1 (KChIP1), represent another class of β-like auxiliary subunits that primarily associate with Kv4 (Shal-type) α subunits to modulate A-type transient outward currents. KCNIP1 has been detected in mouse β-cells and insulinoma cell lines (e.g., MIN6 and RIN-m5F), where it is highly expressed alongside Kv4.1 (Kcnd1) (27, 41). Loss-of-function studies, including siRNA-mediated silencing or CRISPR-based knockdown of KCNIP1, have consistently shown a twofold increase in GSIS compared with controls in these cell models, without affecting insulin gene transcription (98). This enhancement is glucose-dependent, suggesting that KCNIP1 normally acts to limit secretion under stimulatory conditions.

Two primary hypotheses explain these effects. First, KCNIP1, as a Ca²^+^-binding protein, may function as a Ca²^+^ scavenger, buffering intracellular Ca²^+^ increases during depolarization and thereby reducing the Ca²^+^-dependent exocytosis of insulin granules (65). Second, by modulating Kv4 channel kinetics—such as accelerating recovery from inactivation or shifting activation thresholds—KCNIP1 could enhance K^+^ efflux, promoting faster repolarization and limiting the duration of action potentials, which in turn curtails Ca²^+^ entry through voltage-dependent Ca²^+^ channels (28, 41). Knockdown of KCNIP1 would thus reduce these K^+^ currents, delay repolarization, prolong Ca²^+^ signaling, and stimulate GSIS. Although Kv4 channels contribute less to the total Kv current than Kv2.1 in β-cells, their modulation by KCNIP1 highlights a role for β subunits in integrating Ca²^+^ and voltage signals.

Silent α subunits (e.g., Kv6.2 and Kv9.3), which do not form functional homotetramers but heteromerize with active α subunits like Kv2.1, can also be considered β-like modulators in β-cells (38). These subunits attenuate current amplitude, alter inactivation kinetics, or modify voltage dependence, potentially influencing the repolarizing phase and GSIS (37). For example, Kv9.3 expression in β-cells may dampen Kv2.1 activity, and disruptions could mimic β subunit loss by enhancing excitability.

Overall, whereas β subunits are not as pivotal as α subunits in the basal regulation of insulin secretion—evidenced by the dominance of Kv2.1 in repolarization—their modulatory effects on channel diversity and responsiveness to metabolic cues suggest they contribute to β-cell adaptability. Further studies, including in human islets, are needed to clarify their therapeutic potential, such as targeting KCNIP1 to enhance GSIS in type 2 diabetes.

## 9 Pharmacology of Kv channels in β-cells

Insulin secretion alterations rely on the balance of depolarizing and repolarizing currents in the β-cell. K_ATP_, Kv, and K_Ca_ channels collectively determine membrane potential changes; they ultimately influence bursting and Ca²^+^ oscillations. The accumulation of action potentials induces glucose-dependent changes in calcium fluctuations, ultimately triggering islet insulin secretion. Kv channel blockade can increase action potential accumulation and prolong GSIS (99). Kv channels are crucial regulators of membrane excitability and repolarization in excitable cells, including pancreatic β-cells (100, 101).

In the context of GSIS, Kv channels, particularly the Kv2.1 subtype, modulate action potential duration and thereby influence calcium influx through voltage-dependent calcium channels. This, in turn, affects the amount of insulin secreted in response to glucose. Pharmacologically, Kv channels are amenable to modulation by a range of small molecules, peptides, and toxins. Selective Kv channel inhibitors such as tetraethylammonium (TEA) (102), 4-aminopyridine (4-AP) (103–105), and stromatoxin-1 (ScTx1) have been widely used to dissect their physiological functions in β-cells (106–108). TEA and 4-AP, while not subtype-specific, can block Kv currents and potentiate insulin secretion by prolonging β-cells action potentials. More selective blockers like guangxitoxin-1E (GxTx1E) target Kv2.1 channels specifically and have been shown to enhance first-phase insulin secretion without significantly affecting basal insulin release, making them promising candidates for therapeutic development in diabetes (109). Kv channels also show sensitivity to modulation by cellular metabolites and second messengers (110). For instance, protein kinases such as PKA and PKC can phosphorylate Kv2.1, altering its gating kinetics and surface expression. Furthermore, oxidative stress and reactive oxygen species (ROS) may modulate Kv channel activity, linking metabolic state to channel function (110).

Despite these pharmacological insights, no drugs specifically targeting Kv channels have been approved for clinical use in T2DM as of 2025. This absence stems largely from challenges in achieving β-cell-specific modulation. Kv channels, particularly subtypes like Kv2.1, are ubiquitously expressed across various tissues, including neurons, cardiac myocytes, smooth muscle cells, and other excitable and non-excitable cells. Systemic inhibition could therefore elicit off-target effects, such as neurological disturbances (e.g., seizures or altered cognition due to neuronal Kv blockade), cardiovascular complications (e.g., arrhythmias from cardiac Kv disruption), or vascular tone alterations. The lack of subtype- and tissue-selective inhibitors has hindered drug development, as broad-spectrum agents like TEA or 4-AP lack the precision needed for safe therapeutic application. While selective toxins like GxTx-1E show promise in preclinical models by enhancing insulin sensitivity and secretion without widespread side effects, translating these to clinically viable drugs requires overcoming hurdles in delivery, stability, and specificity. Efforts to design small-molecule modulators with improved selectivity continue, but the pleiotropic roles of Kv channels remain a significant barrier.

Elucidating the pharmacology of Kv channels not only deepens our understanding of β-cell electrophysiology but also paves the way for novel therapeutic strategies in T2DM. For example, ubiquitous expression (**Table 2**) risks cardiac (Kv11.1/LQTS) and neuronal side effects; human β-cells rely more on Kv2.2 than Kv2.1. Selective Kv modulators could emerge as insulinotropic agents with a favorable profile-glucose-dependent action potentially reducing hypoglycemia risk compared with KATP-targeted sulfonylureas—provided specificity challenges are addressed.

## 10 Pathological contribution of Kv channels in diabetes

Telmisartan as antihypertensive drugs is one of the angiotensin II types 1 (AT1) receptor blockers (ARBs), can directly inhibit the Kv2.1 channel *in vitro*, and can also result in lower blood glucose and increased plasma insulin concentration in OGTT both in *db/db* mice and in humans. Telmisartan does not enhance insulin secretion under low glucose (2.8 mM) conditions, which means it may be a “smart”-glucose sensor and will not lead to side effects like hyperinsulinemia (111).

Maturation or function genes for more susceptibility to the T2D are identified, which mainly includes more than 40 genomic loci (112). Early study mapped human KCNA7 (Kv1.7) and KCNC3 (Kv3.3) to a region chromosome 19 (19q13.3-13.4) which contains a diabetes susceptibility locus (113). Evidence suggested that the mutation in the KCNQ1 gene is responsible for long QT-syndrome 1 (LQT1), and subjects are more likely to develop hypoglycemia than control subjects (114). In addition, the Kv7.1 channel regulates IKs, which may respond to insulin (115). In a comparative study of Japanese T2D donors with and without insulin therapy, the former indicated a more prolonged corrected QT interval (QTc), suggesting that IKs may be weakened under higher insulin concentrations. As mentioned above, a lower expression of KCNQ1 in β-cells may not contribute to K^+^ current, so its channel function is still little known. It may result in an enhanced expression of the cyclin-dependent kinase inhibitor 1C (Cdkn1c), a cell-cycle inhibitor mouse β-cell, which is responsible for reduced β-cell mass and increased risk of T2D (93). In accordance with this, the weight of Kv1.3 knockout mice is significantly less than that of mice in the control group in response to a high-fat diet, suggesting that this channel may be resistant to obesity which is a characteristic of developing T2D (84). ERG channels including KCNH2 (Kv11.1) have been discovered to be associated with long QT syndrome. Mutation in KCNH2 (Kv11.1) causes long-QT syndrome type 2 (LQT2) due to dysfunction of the pore formation of the α-subunit of the Kv11.1 channel, which prolonged the repolarization in cardiac cells. KCNH2 knockdown in β-cells obtained from patients with LQT2 demonstrated a significant increase in insulin secretion, suggesting that these subjects are prone to develop hypoglycemia (116). An investigation of multigeneration of diabetic models showed that dysfunction of KCNH6 (Kv11.2) played important roles in the development of diabetes diseases from children to adults. Mutation in KCNH6 indicated super-high insulin secretion in newborns, suggesting that it is a suspected cause of hypoglycemia and hyperinsulinemia in the short term. With age in adults, mutation in KCNH6 initially indicated overstimulation of insulin release resulting in endoplasmic reticulum (ER) stress and apoptosis, β-cell mass decrease, and subsequent insulin secretion deficiency, suggesting that dysfunction of KCNH6 is prone to develop hyperglycemia and diabetes in the long term (117).

KCNIP1 has been identified as a T2D-susceptible gene through copy number variations (CNVs). As mentioned earlier, dysfunction of KCNIP1 contributes to impaired β-cell insulin secretion, but the precise mechanisms by which it predisposes to T2D remain incompletely understood. One possibility is that KCNIP1 is responsible for prolonging the action potential, leading to intracellular calcium accumulation that exerts toxic effects and ultimately results in β-cell death (65).

Patients with T2D in the early stages often manifest dysfunction in the first phase of GSIS (118). consisting of a fast first phase followed by a slower second phase. Kv2.1 clusters serve as a reserve pool and bind syntaxin-3 (SYN-3) through their C-terminus, thereby distributing secretory granules to replenish the pool of newcomer secretory granules after glucose stimulation (119). T2D-associated genes are updated in this recent research article (120).

This process is crucial, as newcomer secretory granules contribute to both the first and second phases of GSIS (121). Furthermore, T2D islets show a significant reduction in SNARE and Kv2.1 expression compared with non-diabetic islets. This reduction may partly explain the decrease in Kv2.1 clusters and insufficient binding to SNAREs, which leads to impaired secretory granule reservoir function.

## 11 Conclusion and prospects

The properties of Kv channels in pancreatic β-cells have attracted considerable attention, as the insulinotropic effect of Kv channel inhibition is glucose-dependent. The diversity of Kv channels confers varied biophysical properties, and their precise roles in insulin secretion remain incompletely understood. Inhibition of Kv currents can prolong action potentials and thereby enhance GSIS, particularly through the Kv2.1 channel, which plays a profound role in regulating GSIS. Emerging evidence also supports interactions between SNAREs and Kv2.1 channels in regulating insulin exocytosis and release. Further investigation of pharmacological inhibitors targeting Kv channels may constitute a stepping stone toward T2D treatment. Selective Kv2.1 inhibitors are investigated, exploiting non-electrical SNARE-clustering function, β-cell-specific delivery, and dual Kv2/Kv11 modulators. New 2025 data on KCNH6 regulating mitochondrial complex I and insulin secretion provide additional metabolic therapeutic angles but also highlight long-term β-cell stress risks.

This review elucidates the diverse structures and molecular functions of Kv channels in GSIS, discusses the pharmacology of Kv channel inhibitors and their effects on insulin secretion and explores their therapeutic potential alongside the challenges for future clinical application in T2DM.

## References

[1] Singh A Shadangi S Gupta PK Rana S . Type 2 diabetes mellitus: A comprehensive review of pathophysiology, comorbidities, and emerging therapies. Compr Physiol. (2025) 15(1):e70003. doi: 10.1002/cph4.70003. PMID: 39980164

[2] Kwon YJ Kwon GE Lee HS Choi MH Lee JW . The effect of orlistat on sterol metabolism in obese patients. Front Endocrinol (Lausanne). (2022) 13:824269. doi: 10.3389/fendo.2022.824269. PMID: 35282441 PMC8905288

[3] Heck AM Yanovski JA Calis KA . Orlistat, a new lipase inhibitor for the management of obesity. Pharmacotherapy. (2000) 20:270–9. doi: 10.1592/phco.20.4.270.34882. PMID: 10730683 PMC6145169

[4] Jenner P . Istradefylline, a novel adenosine A2a receptor antagonist, for the treatment of Parkinson's disease. Expert Opin Investig Drugs. (2005) 14:729–38. doi: 10.1517/13543784.14.6.729. PMID: 16004599

[5] Cosentino G Conrad AO Uwaifo GI . Phentermine and topiramate for the management of obesity: A review. Drug Des Dev Ther. (2013) 7:267–78. doi: 10.2147/Dddt.S31443. PMID: 23630412 PMC3623549

[6] Jensen . Effect of orlistat on weight and body composition in obese adolescents: A randomized controlled trial (Vol 293, Pg 2873, 2005). JAMA J Am Med Assoc. (2005) 294:1491. doi: 10.1016/j.accreview.2005.08.191. PMID: 15956632

[7] le Roux CW Fils-Aimé N Camacho F Gould E Barakat M . The relationship between early weight loss and weight loss maintenance with naltrexone-bupropion therapy. Eclinicalmedicine. (2022) 49:101436. doi: 10.1016/j.eclinm.2022.101436. PMID: 35747175 PMC9156890

[8] Christou GA Kiortsis DN . The efficacy and safety of the naltrexone/bupropion combination for the treatment of obesity: An update. Horm-Int J Endocrino. (2015) 14:370–5. doi: 10.14310/horm.2002.1600. PMID: 26188223

[9] Wang GJ Tomasi D Volkow ND Wang R Telang F Caparelli EC . Effect of combined naltrexone and bupropion therapy on the brain's reactivity to food cues. Int J Obes. (2014) 38:682–8. doi: 10.1038/ijo.2013.145. PMID: 23924756 PMC4010969

[10] Alruwaili H Dehestani B le Roux CW . Clinical impact of liraglutide as a treatment of obesity. Clin Pharmacol-Adv A. (2021) 13:53–60. doi: 10.2147/Cpaa.S276085. PMID: 33732030 PMC7958997

[11] Mehta A Marso SP Neeland IJ . Liraglutide for weight management: A critical review of the evidence. Obes Sci Pract. (2017) 3:3–14. doi: 10.1002/osp4.84. PMID: 28392927 PMC5358074

[12] Tamayo-Trujillo R Ruiz-Pozo VA Cadena-Ullauri S Guevara-Ramírez P Paz-Cruz E Zambrano-Villacres R . Molecular mechanisms of semaglutide and liraglutide as a therapeutic option for obesity. Front Nutr. (2024) 11:1398059. doi: 10.3389/fnut.2024.1398059. PMID: 38742021 PMC11090168

[13] Alorfi NM Algarni AS . Clinical impact of semaglutide, a glucagon-like peptide-1 receptor agonist, on obesity management: A review. Clin Pharmacol-Adv A. (2022) 14:61–7. doi: 10.2147/Cpaa.S374741. PMID: 35958046 PMC9357557

[14] Kesavadev J Shankar A Joshi SR Ashik A Yasmin S Basanth A . How effective is weight loss by oral semaglutide on glycemic control in a real-world setting? Diabetes. (2024) 73(Suppl 1):803-P. doi: 10.2337/db24-803-P

[15] Barbosa BF de Moraes FCA Barbosa CB Santos PTKP da Silva IP da Silva BAA . Efficacy and safety of setmelanotide, a melanocortin-4 receptor agonist, for obese patients: A systematic review and meta-analysis. J Pers Med. (2023) 13:1460. doi: 10.3390/jpm13101460. PMID: 37888071 PMC10608339

[16] Qamar S Mallik R Makaronidis J . Setmelanotide: A melanocortin-4 receptor agonist for the treatment of severe obesity due to hypothalamic dysfunction. touchREV Endocrinol. (2024) 20:62–71. doi: 10.17925/EE.2024.20.2.9. PMID: 39526054 PMC11548362

[17] De Block C Bailey C Wysham C Hemmingway A Allen SE Peleshok J . Tirzepatide for the treatment of adults with type 2 diabetes: An endocrine perspective. Diabetes Obes Metab. (2023) 25:3–17. doi: 10.1111/dom.14831. PMID: 35929488 PMC10087310

[18] Cai WT Zhang RB Yao Y Wu QH Zhang JP . Tirzepatide as a novel effective and safe strategy for treating obesity: A systematic review and meta-analysis of randomized controlled trials. Front Public Health. (2024) 12:1277113. doi: 10.3389/fpubh.2024.1277113. PMID: 38356942 PMC10864442

[19] Federation ID . Idf diabetes atlas, 10th edn. Brussels, Belgium: International Diabetes Federation (2021).

[20] DeFronzo RA Ferrannini E Groop L Henry RR Herman WH Holst JJ . Type 2 diabetes mellitus. Nat Rev Dis Primers. (2015) 1:1–22. doi: 10.1007/978-3-319-27317-4_8-1 27189025

[21] Zimmerman BR . Sulfonylureas. Endocrinol Metab Clinics North America. (1997) 26:511–22. doi: 10.1016/s0889-8529(05)70264-4. PMID: 9314012

[22] Proks P Reimann F Green N Gribble F Ashcroft F . Sulfonylurea stimulation of insulin secretion. Diabetes. (2002) 51:S368–76. doi: 10.2337/diabetes.51.2007.s368. PMID: 12475777

[23] Meissner HP Schmeer W . The significance of calcium ions for the glucose–induced electrical activity of pancreatic B–cells. In: Ohnishi ST Endo M , editors. The Mechanism of Gated Calcium Transport Across Biological Membranes. New York: Academic Press / Elsevier (1981). p. 157–65.

[24] Alam KA Svalastoga P Martinez A Glennon JC Haavik J . Potassium channels in behavioral brain disorders. Molecular mechanisms and therapeutic potential: A narrative review. Neurosci Biobehav R. (2023) 152:105301. doi: 10.1016/j.neubiorev.2023.105301. PMID: 37414376

[25] Fu J Dai X Plummer G Suzuki K Bautista A Githaka JM . Kv2. 1 clustering contributes to insulin exocytosis and rescues human B-cell dysfunction. Diabetes. (2017) 66:1890–900. doi: 10.2337/db16-1170. PMID: 28607108 PMC5482075

[26] Lingle CJ Martinez-Espinosa PL Guarina L Carbone E . Roles of Na+, Ca2+, and K+ channels in the generation of repetitive firing and rhythmic bursting in adrenal chromaffin cells. Pflugers Arch. (2018) 470:39–52. doi: 10.1007/s00424-017-2048-1. PMID: 28776261 PMC5765858

[27] Xiao Y Yang J Ji W He Q Mao L Shu Y . A- and D-type potassium currents regulate axonal action potential repolarization in midbrain dopamine neurons. Neuropharmacology. (2021) 185:108399. doi: 10.1016/j.neuropharm.2020.108399. PMID: 33400937

[28] Herrington J Zhou YP Bugianesi RM Dulski PM Feng Y Warren VA . Blockers of the delayed-rectifier potassium current in pancreatic B-cells enhance glucose-dependent insulin secretion. Diabetes. (2006) 55:1034–42. doi: 10.2337/diabetes.55.04.06.db05-0788. PMID: 16567526

[29] Zhao MM Lu J Li S Wang H Cao X Li Q . Berberine is an insulin secretagogue targeting the Kcnh6 potassium channel. Nat Commun. (2021) 12:1–14. doi: 10.1038/s41467-021-25952-2. PMID: 34556670 PMC8460738

[30] Li Y Um SY McDonald TV . Voltage-gated potassium channels: Regulation by accessory subunits. Neuroscientist. (2006) 12:199–210. doi: 10.1177/1073858406287717. PMID: 16684966

[31] Bocksteins E Snyders DJ . Electrically silent Kv subunits: Their molecular and functional characteristics. Physiology. (2012) 27:73–84. doi: 10.1152/physiol.00023.2011. PMID: 22505664

[32] Bezanilla F . The voltage sensor in voltage-dependent ion channels. Physiol Rev. (2000) 80:555–92. doi: 10.1152/physrev.2000.80.2.555. PMID: 10747201

[33] Heginbotham L Lu Z Abramson T MacKinnon R . Mutations in the K+ channel signature sequence. Biophys J. (1994) 66:1061–7. doi: 10.1016/s0006-3495(94)80887-2. PMID: 8038378 PMC1275813

[34] Bocksteins E . Kv5, Kv6, Kv8, and Kv9 subunits: No simple silent bystanders. J Gen Physiol. (2016) 147:105–25. doi: 10.1085/jgp.201511507. PMID: 26755771 PMC4727947

[35] Ferns M van der List D Vierra NC Lacey T Murray K Kirmiz M . Electrically silent Kvs subunits associate with native Kv2 channels in brain and impact diverse properties of channel function. bioRxiv. (2024). doi: 10.1101/2024.01.25.577135. PMID: 38328147 PMC10849721

[36] Xu Z Khan S Schnicker NJ Baker S . Pentameric assembly of the Kv2.1 tetramerization domain. Acta Crystallogr D. (2022) 78:792–802. doi: 10.1107/S205979832200568x. PMID: 35647925 PMC9159280

[37] Hugnot JP Salinas M Lesage F Guillemare E De Weille J Heurteaux C . Kv8. 1, a new neuronal potassium channel subunit with specific inhibitory properties towards Shab and Shaw channels. EMBO J. (1996) 15:3322–31. doi: 10.1002/j.1460-2075.1996.tb00697.x. PMID: 8670833 PMC451895

[38] Isacoff EY Jan YN Jan LY . Evidence for the formation of heteromultimeric potassium channels in Xenopus oocytes. Nature. (1990) 345:530–4. doi: 10.1038/345530a0. PMID: 2112229

[39] Vicente R Escalada A Villalonga N Texido L Roura-Ferrer M Martín-Satué M . Association of Kv1. 5 and Kv1. 3 contributes to the major voltage-dependent K+ channel in macrophages. J Biol Chem. (2006) 281:37675–85. doi: 10.1074/jbc.m605617200. PMID: 17038323

[40] Salkoff L . Genetic and voltage-clamp analysis of a Drosophila potassium channel. In: Cold Spring Harbor Symposia on Quantitative Biology. Vol. 48. Salkoff L , editor. Cold Spring Harbor, New York: Cold Spring Harbor Laboratory Press (1983) 221–31. 10.1101/sqb.1983.048.01.025 6327157

[41] Swartz KJ . Towards a structural view of gating in potassium channels. Nat Rev Neurosci. (2004) 5:905–16. doi: 10.1038/nrn1559. PMID: 15550946

[42] Bezanilla F . Ion channels: From conductance to structure. Neuron. (2008) 60:456–68. doi: 10.1016/j.neuron.2008.10.035. PMID: 18995820

[43] Bezanilla F . How membrane proteins sense voltage. Nat Rev Mol Cell Bio. (2008) 9:323–32. doi: 10.1038/nrm2376. PMID: 18354422

[44] Beckh S Pongs O . Members of the Rck potassium channel family are differentially expressed in the rat nervous system. EMBO J. (1990) 9:777–82. doi: 10.1002/j.1460-2075.1990.tb08173.x. PMID: 2311579 PMC551736

[45] Klumpp DJ Farber DB Bowes C Song EJ Pinto LH . The potassium channel Mbk1 (Kv1. 1) is expressed in the mouse retina. Cell Mol Neurobiol. (1991) 11:611–22. doi: 10.1007/bf00741449. PMID: 1723658 PMC11567379

[46] Racapé J Lecoq A Romi-Lebrun R Liu J Kohler M Garcia ML . Characterization of a novel radiolabeled peptide selective for a subpopulation of voltage-gated potassium channels in mammalian brain. J Biol Chem. (2002) 277:3886–93. doi: 10.1074/jbc.M107766200, PMID: 11707459

[47] Roberds SL Tamkun MM . Cloning and tissue-specific expression of five voltage-gated potassium channel cdnas expressed in rat heart. Proc Natl Acad Sci. (1991) 88:1798–802. doi: 10.1073/pnas.88.5.1798. PMID: 1705709 PMC51112

[48] Tsaur M-L Sheng M Lowenstein DH Jan YN Jan LY . Differential expression of K+ channel mrnas in the rat brain and down-regulation in the hippocampus following seizures. Neuron. (1992) 8:1055–67. doi: 10.1016/0896-6273(92)90127-y. PMID: 1610565

[49] Gutman GA Chandy KG Grissmer S Lazdunski M Mckinnon D Pardo LA . International union of pharmacology. Liii. Nomenclature and molecular relationships of voltage-gated potassium channels. Pharmacol Rev. (2005) 57:473–508. doi: 10.1124/pr.57.4.10. PMID: 16382104

[50] Wulff H Calabresi PA Allie R Yun S Pennington M Beeton C . The voltage-gated Kv1. 3 K+ channel in effector memory T cells as new target for Ms. J Clin Invest. (2003) 111:1703–13. doi: 10.1172/jci200316921e. PMID: 12782673 PMC156104

[51] Wulff H Knaus H-G Pennington M Chandy KG . K+ channel expression during B cell differentiation: Implications for immunomodulation and autoimmunity. J Immunol. (2004) 173:776–86. doi: 10.4049/jimmunol.173.2.776. PMID: 15240664

[52] Ravens U Wettwer E . Ultra-rapid delayed rectifier channels: Molecular basis and therapeutic implications. Cardiovasc Res. (2011) 89:776–85. doi: 10.1093/cvr/cvq398. PMID: 21159668

[53] Jacobson DA Kuznetsov A Lopez JP Kash S Ämmälä CE Philipson LH . Kv2. 1 ablation alters glucose-induced islet electrical activity, enhancing insulin secretion. Cell Metab. (2007) 6:229–35. doi: 10.1016/j.cmet.2007.07.010. PMID: 17767909 PMC2699758

[54] Li XN Herrington J Petrov A Ge L Eiermann G Xiong Y . The role of voltage-gated potassium channels Kv2. 1 and Kv2. 2 in the regulation of insulin and somatostatin release from pancreatic islets. J Pharmacol Exp Ther. (2013) 344:407–16. doi: 10.1124/jpet.112.199083. PMID: 23161216

[55] Birnbaum SG Varga AW Yuan L-L Anderson AE Sweatt JD Schrader LA . Structure and function of Kv4-family transient potassium channels. Physiol Rev. (2004) 84:803–33. doi: 10.1152/physrev.00039.2003. PMID: 15269337

[56] Biel M Zong X Ludwig A Sautter A Hofmann F . Molecular cloning and expression of a modulatory subunit of the cyclic nucleotide-gated cation channel (∗). J Biol Chem. (1996) 271:6349–55. doi: 10.1074/jbc.271.11.6349. PMID: 8626431

[57] Robbins J . Kcnq potassium channels: Physiology, pathophysiology, and pharmacology. Pharmacol Ther. (2001) 90:1–19. doi: 10.1016/s0163-7258(01)00116-4. PMID: 11448722

[58] Su C-C Li S-Y Yang J-J Su M-C Lin M-J . Studies of the effect of ionomycin on the Kcnq4 channel expressed in Xenopus oocytes. Biochem Biophys Res Commun. (2006) 348:295–300. doi: 10.1016/j.bbrc.2006.07.053. PMID: 16876114

[59] Yang J-K Lu J Yuan S-S Cao X Qiu H-Y Shi T-T . From hyper-to hypoinsulinemia and diabetes: Effect of Kcnh6 on insulin secretion. Cell Rep. (2018) 25:3800–10. doi: 10.1016/j.celrep.2018.12.005. PMID: 30590050

[60] MacDonald P Wheeler M . Voltage-dependent K+ channels in pancreatic beta cells: Role, regulation and potential as therapeutic targets. Diabetologia. (2003) 46:1046–62. doi: 10.1007/s00125-003-1159-8. PMID: 12830383

[61] Rorsman P Ashcroft FM . Pancreatic B-cell electrical activity and insulin secretion: Of mice and men. Physiol Rev. (2018) 98:117–214. doi: 10.1152/physrev.00008.2017. PMID: 29212789 PMC5866358

[62] Smith PA Bokvist K Rorsman P . Demonstration of a-currents in pancreatic islet cells. Pflügers Archiv. (1989) 413:441–3. doi: 10.1007/bf00584497. PMID: 2648325

[63] Ma Z Lavebratt C Almgren M Portwood N Forsberg LE Bränström R . Evidence for presence and functional effects of Kv1. 1 channels in β-cells: General survey and results from Mceph/Mceph mice. PloS One. (2011) 6:e18213. doi: 10.1371/journal.pone.0018213. PMID: 21483673 PMC3071710

[64] Finol‐Urdaneta RK Remedi MS Raasch W Becker S Clark RB Strüver N . Block of Kv1. 7 potassium currents increases glucose‐stimulated insulin secretion. EMBO Mol Med. (2012) 4:424–34. doi: 10.1002/emmm.201200218. PMID: 22438204 PMC3403299

[65] MacDonald PE Sewing S Wang J Joseph JW Smukler SR Sakellaropoulos G . Inhibition of Kv2. 1 voltage-dependent K+ channels in pancreatic β-cells enhances glucose-dependent insulin secretion. J Biol Chem. (2002) 277:44938–45. doi: 10.1074/jbc.m205532200. PMID: 12270920

[66] Roe MW Worley JF Mittal AA Kuznetsov A DasGupta S Mertz RJ . Expression and function of pancreatic β-cell delayed rectifier K+ channels: Role in stimulus-secretion coupling. J Biol Chem. (1996) 271:32241–6. doi: 10.1074/jbc.271.50.32241. PMID: 8943282

[67] Yamagata K Senokuchi T Lu M Takemoto M Karim MF Go C . Voltage-gated K+ channel Kcnq1 regulates insulin secretion in Min6 β-cell line. Biochem Biophys Res Commun. (2011) 407:620–5. doi: 10.1016/j.bbrc.2011.03.083. PMID: 21426901

[68] Skelin Klemen M Dolenšek J Slak Rupnik M Stožer A . The triggering pathway to insulin secretion: Functional similarities and differences between the human and the mouse β cells and their translational relevance. Islets. (2017) 9:109–39. doi: 10.1080/19382014.2017.1342022. PMID: 28662366 PMC5710702

[69] Braun M Ramracheya R Bengtsson M Zhang Q Karanauskaite J Partridge C . Voltage-gated ion channels in human pancreatic B-cells: Electrophysiological characterization and role in insulin secretion. Diabetes. (2008) 57:1618–28. doi: 10.2337/db07-0991. PMID: 18390794

[70] Yan L Figueroa DJ Austin CP Liu Y Bugianesi RM Slaughter RS . Expression of voltage-gated potassium channels in human and rhesus pancreatic islets. Diabetes. (2004) 53:597–607. doi: 10.2337/diabetes.53.3.597. PMID: 14988243

[71] Yang SN Shi Y Yang G Li Y Yu J Berggren PO . Ionic mechanisms in pancreatic B cell signaling. Cell Mol Life Sci. (2014) 71:4149–77. doi: 10.1007/s00018-014-1680-6. PMID: 25052376 PMC11113777

[72] Henquin JC . Regulation of insulin secretion: A matter of phase control and amplitude modulation. Diabetologia. (2009) 52:739–51. doi: 10.1007/s00125-009-1314-y. PMID: 19288076

[73] Cook DL Hales N . Intracellular Atp directly blocks K+ channels in pancreatic B-cells. Nature. (1984) 311:271–3. doi: 10.1038/311271a0. PMID: 6090930

[74] Gaisano HY . Recent new insights into the role of Snare and associated proteins in insulin granule exocytosis. Diabetes Obes Metab. (2017) 19:115–23. doi: 10.1111/dom.13001. PMID: 28880475

[75] Pan C Liu Y Wang L Fan WY Ni Y Zhang X . The Kv2.2 channel mediates the inhibition of prostaglandin E2 on glucose-stimulated insulin secretion in pancreatic beta-cells. Elife. (2025) 13:RP97234. doi: 10.7554/eLife.97234. PMID: 40028769 PMC11875535

[76] Wang H Li Q Yuan YC Han XC Cao YT Yang JK . Kcnh6 channel promotes insulin exocytosis via interaction with Munc18–1 independent of electrophysiological processes. Cell Mol Life Sci. (2024) 81:86. doi: 10.1007/s00018-024-05134-1. PMID: 38349432 PMC10864572

[77] Geoffrey WA . Kv channel ancillary subunits: Where do we go from here? Physiol (Bethesda). (2022) 37:225–41. doi: 10.1152/physiol.00005.2022. PMID: 35797055 PMC9394777

[78] Ottschytsch N Raes A Van Hoorick D Snyders DJ . Obligatory heterotetramerization of three previously uncharacterized Kv channel A-subunits identified in the human genome. P Natl Acad Sci USA. (2002) 99:7986–91. doi: 10.1073/pnas.122617999. PMID: 12060745 PMC123007

[79] Salinas M Duprat F Heurteaux C Hugnot JP Lazdunski M . New modulatory alpha subunits for mammalian Shab K+ channels. J Biol Chem. (1997) 272:24371–9. doi: 10.1074/jbc.272.39.24371. PMID: 9305895

[80] Stocker M Kerschensteiner D . Cloning and tissue distribution of two new potassium channel A-subunits from rat brain. Biochem Bioph Res Co. (1998) 248:927–34. doi: 10.1006/bbrc.1998.9072. PMID: 9704029

[81] Al-Sabi A Shamotienko O Dhochartaigh SN Muniyappa N Le Berre M Shaban H . Arrangement of Kv1 A subunits dictates sensitivity to tetraethylammonium. J Gen Physiol. (2010) 136:273–82. doi: 10.1085/jgp.200910398. PMID: 20805574 PMC2931144

[82] Bean BP . The action potential in mammalian central neurons. Nat Rev Neurosci. (2007) 8:451–65. doi: 10.1038/nrn2148. PMID: 17514198

[83] Robbins CA Tempel BL . Kv1. 1 and Kv1. 2: Similar channels, different seizure models. Epilepsia. (2012) 53:134–41. doi: 10.1111/j.1528-1167.2012.03484.x. PMID: 22612818

[84] Xu J Koni PA Wang P Li G Kaczmarek L Wu Y . The voltage-gated potassium channel Kv1. 3 regulates energy homeostasis and body weight. Hum Mol Genet. (2003) 12:551–9. doi: 10.1093/hmg/ddg049. PMID: 12588802

[85] Xu J Wang P Li Y Li G Kaczmarek LK Wu Y . The voltage-gated potassium channel Kv1. 3 regulates peripheral insulin sensitivity. Proc Natl Acad Sci. (2004) 101:3112–7. doi: 10.1073/pnas.0308450100. PMID: 14981264 PMC365752

[86] Wolf-Goldberg T Michaelevski I Sheu L Gaisano HY Chikvashvili D Lotan I . Target soluble N-ethylmaleimide-sensitive factor attachment protein receptors (T-snares) differently regulate activation and inactivation gating of Kv2. 2 and Kv2. 1: Implications on pancreatic islet cell Kv channels. Mol Pharmacol. (2006) 70:818–28. doi: 10.1124/mol.105.021717. PMID: 16754785

[87] Hayashi M Wang J Hede SE Novak I . An intermediate-conductance Ca2+-activated K+ channel is important for secretion in pancreatic duct cells. Am J Physiol Cell Physiol. (2012) 303:C151–9. doi: 10.1152/ajpcell.00089.2012. PMID: 22555847

[88] Liu L Wang F Lu H Ren X Zou J . Chromanol 293b, an inhibitor of Kcnq1 channels, enhances glucose-stimulated insulin secretion and increases glucagon-like peptide-1 level in mice. Islets. (2014) 6:e962386. doi: 10.4161/19382014.2014.962386. PMID: 25437377 PMC4588556

[89] Torekov SS Iepsen E Christiansen M Linneberg A Pedersen O Holst JJ . Kcnq1 long QT syndrome patients have hyperinsulinemia and symptomatic hypoglycemia. Diabetes. (2014) 63:1315–25. doi: 10.2337/db13-1454. PMID: 24357532

[90] Lubberding AF Juhl CR Skovhoj EZ Kanters JK Mandrup-Poulsen T Torekov SS . Celebrities in the heart, strangers in the pancreatic beta cell: Voltage-gated potassium channels K(V) 7.1 and K(V) 11.1 bridge long QT syndrome with hyperinsulinaemia as well as type 2 diabetes. Acta Physiol (Oxf). (2022) 234:e13781. doi: 10.1111/apha.13781. PMID: 34990074 PMC9286829

[91] Drewe J Verma S Frech G Joho R . Distinct spatial and temporal expression patterns of K+ channel mRNAs from different subfamilies. J Neurosci. (1992) 12:538–48. doi: 10.1523/jneurosci.12-02-00538.1992. PMID: 1740690 PMC6575610

[92] Thompson E Eldstrom J Fedida D . Hormonal signaling actions on Kv7. 1 (Kcnq1) channels. Annu Rev Pharmacol Toxicol. (2021) 61:381–400. doi: 10.1146/annurev-pharmtox-010919-023645. PMID: 32667860

[93] Asahara S Etoh H Inoue H Teruyama K Shibutani Y Ihara Y . Paternal allelic mutation at the Kcnq1 locus reduces pancreatic β-cell mass by epigenetic modification of Cdkn1c. Proc Natl Acad Sci. (2015) 112:8332–7. doi: 10.1073/pnas.1422104112. PMID: 26100882 PMC4500236

[94] Rosengren AH Braun M Mahdi T Andersson SA Travers ME Shigeto M . Reduced insulin exocytosis in human pancreatic β-cells with gene variants linked to type 2 diabetes. Diabetes. (2012) 61:1726–33. doi: 10.2337/db11-1516. PMID: 22492527 PMC3379663

[95] Mühlbauer E Bazwinsky I Wolgast S Klemenz A Peschke E . Circadian changes of ether-a-go-go-related-gene (Erg) potassium channel transcripts in the rat pancreas and β-cell. Cell Mol Life Sci. (2007) 64:768–80. doi: 10.1007/s00018-007-6448-9, PMID: 17322986 PMC11138461

[96] Blodgett DM Nowosielska A Afik S Pechhold S Cura AJ Kennedy NJ . Novel observations from next-generation RNA sequencing of highly purified human adult and fetal islet cell subsets. Diabetes. (2015) 64:3172–81. doi: 10.2337/db15-0039. PMID: 25931473 PMC4542439

[97] Rosati B Marchetti P Crociani O Lecchi M Lupi R Arcangeli A . Glucose‐and arginine‐induced insulin secretion by human pancreatic β‐cells: The role of HERG K+ channels in firing and release. FASEB J. (2000) 14:2601–10. doi: 10.1096/fj.00-0077com, PMID: 11099479

[98] Dwenger MM Raph SM Baba SP Moore JB Nystoriak MA . Diversification of potassium currents in excitable cells via Kvβ proteins. Cells. (2022) 11(14):2230. doi: 10.3390/cells11142230. PMID: 35883673 PMC9317154

[99] Philipson LH . β-cell ion channels: Keys to endodermal excitability. Horm Metab Res. (1999) 31:455–61. doi: 10.1055/s-2007-978774. PMID: 10494870

[100] MacDonald PE Ha XF Wang J Smukler SR Sun AM Gaisano HY . Members of the Kv1 and Kv2 voltage-dependent K channel families regulate insulin secretion. Mol Endocrinol. (2001) 15:1423–35. doi: 10.1210/me.15.8.1423. PMID: 11463864

[101] Atwater I Ribalet B Rojas E . Mouse pancreatic beta-cells - Tetraethylammonium blockage of the potassium permeability increase induced by depolarization. J Physiol-London. (1979) 288:561–74. doi: 10.1113/jphysiol.1979.sp012713. PMID: 381635 PMC1281444

[102] Rodríguez-Rangel S Bravin AD Ramos-Torres KM Brugarolas P Sánchez-Rodríguez JE . Structure-activity relationship studies of four novel 4-aminopyridine K channel blockers. Sci Rep-Uk. (2020) 10:52. doi: 10.1038/s41598-019-56245-w. PMID: 31919372 PMC6952366

[103] Armstrong CM Loboda A . A model for 4-aminopyridine action on K channels: Similarities to tetraethylammonium ion action. Biophys J. (2001) 81:895–904. doi: 10.1016/S0006-3495(01)75749-9. PMID: 11463633 PMC1301561

[104] Choquet D Korn H . Mechanism of 4-aminopyridine action on voltage-gated potassium channels in lymphocytes. J Gen Physiol. (1992) 99:217–40. doi: 10.1085/jgp.99.2.217. PMID: 1613484 PMC2216608

[105] Zhang S Stix R Orabi EA Bernhardt N Faraldo-Gomez JD . Distinct mechanisms of inhibition of Kv2 potassium channels by tetraethylammonium and Ry785. bioRxiv. (2024). doi: 10.1101/2024.07.25.605170. PMID: 42043292 PMC13120820

[106] Zhong XZ Abd-Elrahman KS Liao CH El-Yazbi AF Walsh EJ Walsh MP . Stromatoxin-sensitive, heteromultimeric Kv2.1/Kv9.3 channels contribute to myogenic control of cerebral arterial diameter. J Physiol-London. (2010) 588:4519–37. doi: 10.1113/jphysiol.2010.196618. PMID: 20876197 PMC3008855

[107] Jensen MV Haldeman JM Zhang HT Lu DH Huising MO Vale WW . Control of voltage-gated potassium channel Kv2.2 expression by pyruvate-isocitrate cycling regulates glucose-stimulated insulin secretion. J Biol Chem. (2013) 288:23128–40. doi: 10.1074/jbc.M113.491654. PMID: 23788641 PMC3743485

[108] Kusunoki M Hayashi M Shoji T Uba T Tanaka H Sumi C . Propofol inhibits stromatoxin-1-sensitive voltage-dependent K+ channels in pancreatic β-cells and enhances insulin secretion. PeerJ. (2019) 7:e8157. doi: 10.7717/peerj.8157. PMID: 31824770 PMC6894434

[109] Sahoo N Hoshi T Heinemann SH . Oxidative modulation of voltage-gated potassium channels. Antioxid Redox Sign. (2014) 21:933–52. doi: 10.1089/ars.2013.5614. PMID: 24040918 PMC4116129

[110] Liu T Cui L Xue H Yang X Liu M Zhi L . Telmisartan potentiates insulin secretion via ion channels, independent of the At1 receptor and Pparγ. Front Pharmacol. (2021) 12:2471. doi: 10.3389/fphar.2021.739637. PMID: 34594226 PMC8477257

[111] Bonnefond A Froguel P Vaxillaire M . The emerging genetics of type 2 diabetes. Trends Mol Med. (2010) 16:407–16. doi: 10.1016/j.molmed.2010.06.004. PMID: 20728409

[112] Kalman K Nguyen A Tseng-Crank J Dukes ID Chandy G Hustad CM . Genomic organization, chromosomal localization, tissue distribution, and biophysical characterization of a novel mammalianshaker-related voltage-gated potassium channel, Kv1. 7. J Biol Chem. (1998) 273:5851–7. doi: 10.1074/jbc.273.10.5851. PMID: 9488722

[113] Oertli A Rinné S Moss R Kääb S Seemann G Beckmann BM . Molecular mechanism of autosomal recessive long QT-syndrome 1 without deafness. Int J Mol Sci. (2021) 22:1112. doi: 10.3390/ijms22031112. PMID: 33498651 PMC7865240

[114] Zhang J Juhl CR Hylten-Cavallius L Salling-Olsen M Linneberg A Holst JJ . Gain-of-function mutation in the voltage-gated potassium channel gene Kcnq1 and glucose-stimulated hypoinsulinemia-case report. BMC Endocr Disord. (2020) 20:1–5. doi: 10.1186/s12902-020-0513-x. PMID: 32164657 PMC7069191

[115] Hyltén-Cavallius L Iepsen EW Wewer Albrechtsen NJ Svendstrup M Lubberding AF Hartmann B . Patients with long-QT syndrome caused by impaired HERG-encoded Kv11. 1 potassium channel have exaggerated endocrine pancreatic and incretin function associated with reactive hypoglycemia. Circulation. (2017) 135:1705–19. doi: 10.1161/circulationaha.116.024279. PMID: 28235848 PMC5412733

[116] Wible BA Yang Q Kuryshev YA Accili EA Brown AM . Cloning and expression of a novel K+ channel regulatory protein, KChAP. J Biol Chem. (1998) 273:11745–51. doi: 10.1074/jbc.273.19.11745. PMID: 9565597

[117] Lee HS Moon S Yun JH Lee M Hwang MY Kim YJ . Genome-wide copy number variation study reveals KCNIP1 as a modulator of insulin secretion. Genomics. (2014) 104:113–20. doi: 10.1016/j.ygeno.2014.05.004. PMID: 24886904

[118] Cerasi E Efendić S Luft R . Dose-response relation between plasma-insulin and blood-glucose levels during oral glucose loads in prediabetic and diabetic subjects. Lancet. (1973) 301:794–7. doi: 10.1016/s0140-6736(73)90599-0. PMID: 4121221

[119] Greitzer-Antes D Xie L Qin T Xie H Zhu D Dolai S . Kv2. 1 clusters on β-cell plasma membrane act as reservoirs that replenish pools of newcomer insulin granule through their interaction with syntaxin-3. J Biol Chem. (2018) 293:6893–904. doi: 10.1074/jbc.ra118.002703. PMID: 29549124 PMC5936832

[120] Cowan E Karagiannopoulos A Pollastri A Asai A Nagao M Maziarz M . Network-based insights into miRNA regulation of beta-cell insulin secretion in type 2 diabetes. iScience. (2025) 28:114200. doi: 10.1016/j.isci.2025.114200. PMID: 41438030 PMC12721147

[121] Gaisano HY . Here come the newcomer granules, better late than never. Trends Endocrinol Metab. (2014) 25:381–8. doi: 10.1016/j.tem.2014.03.005. PMID: 24746186

